# Attachment styles among students at the faculty of medicine of Sfax and parental separation and divorce

**DOI:** 10.1192/j.eurpsy.2025.1499

**Published:** 2025-08-26

**Authors:** Y. Yaich, A. Bouallegui, N. Ben Hamed, H. Khiari, A. Hakiri, G. Amri, R. Ghachem

**Affiliations:** 1 Psychiatry B, Razi hospital, Tunis, Tunisia

## Abstract

**Introduction:**

The theory of attachment is one of the most influential theories in developmental psychology. It was originally developed by John Bowlby.This theory states that early relational experiences, particularly those with primary attachment figures (usually parents), influence the formation of internal working models that guide social interactions and attitudes toward oneself and others throughout life. This framework is particularly relevant in the context of students at the Faculty of Medicine of Sfax, where the pressures of rigorous academic demands intersect with personal histories that may include parental separation and divorce. Research indicates that parental separation and divorce can negatively impact attachment security, leading individuals with such experiences to have a higher likelihood of developing distrust in relationships.

**Objectives:**

The purpose of this study was to evaluate the prevalence of different attachment styles among medical externs using the RSQ scale and to examine the relationship between these attachment styles and parental separation or divorce.

**Methods:**

This is a cross-sectional, descriptive, and analytical study. It was conducted over a period of five months among students at the Faculty of Medicine of Sfax using an online form that included sociodemographic data, medical history, lifestyle habits and the “Relationship Scale Questionnaire” (RSQ).

**Results:**

The average age was 21.63 years. The distribution of students according to their attachment styles showed that avoidant attachment was the most prevalent (29%, n=150), and women exhibited more ambivalent attachment than men (p=0,031). Ambivalent attachment was significantly associated with sexual orientation (p=0,025). A significant link was demonstrated between parental marital status and avoidant attachment style (p=0,008).Indeed, an avoidant attachment style was more frequently found among students whose parents were separated or divorced. Ambivalent and disorganized attachment styles were associated with separation from the mother during the first five years of life (p=0,004 and p= 0,011) and separation from the father during the first five years of life (p=0,034 and p = 0,010). A correlation was found between repeating a year and avoidant attachment style (p=0,018). Single students were primarily avoidant (p=0,039), while married students showed more secure (p=0,019) and disorganized attachment (p=0,029).

**Conclusions:**

In conclusion, the attachment styles developed in childhood, particularly in the context of parental separation and divorce, have significant implications for students at the Faculty of Medicine of Sfax.By addressing the impact of attachment styles, educators and counselors can better equip students to thrive both personally and professionally, ultimately enhancing their contributions to the medical field.

**Disclosure of Interest:**

None Declared

